# Dr. Henry Clay Hill

**Published:** 1901-03

**Authors:** 


Dr. Henry Clay Hill, of Lockport, died at his home in that city February 8, 1901, aged 68 years. He was a graduate of the University of Michigan in 1859, and served as assistant surgeon from September 10 to December 3, 1862, in Colonel Peter A. Porter's regiment, the 129th New York Volunteers, afterward the 8th New York Heavy Artillery. Dr. Hill located at Lockport in 1877, and continued to reside there until his death.

